# Supplementary material for: Aberrant B Cell Receptor Signaling in Naïve B Cells from Patients with Idiopathic Pulmonary Fibrosis
Source: Cells. 2021 May 26;10(6):1321. doi: 10.3390/cells10061321 (PMC8226954; doi:10.3390/cells10061321)
Supplement: Supplementary file 1 [file cells-10-01321-s001.zip › supplementary.v2.pdf]

cells

MDPI

Supplementary data to:

Aberrant B Cell Receptor Signaling in Naïve B Cells from Patients with Idiopathic Pulmonary Fibrosis

Stefan F. H. Neys, Peter Heukels, Jennifer A. C. van Hulst, Jasper Rip †, Marlies S. Wijsenbeek, Rudi W. Hendriks, *,‡ and Odilia B. J. Corneth *,‡

Department of Pulmonary Medicine, Erasmus University Medical Center, Rotterdam, 3015 GD, The Netherlands; s.neys@erasmusmc.nl (S.F.H.N.); pheukels@amphia.nl (P.H.); j.vanhulst@erasmusmc.nl (J.A.C.v.H.); j.rip@erasmusmc.nl (J.R.); m.wijsenbeek-lourens@erasmusmc.nl (M.S.W.)
* Correspondence: r.hendriks@erasmusmc.nl (R.W.H.); o.corneth@erasmusmc.nl (O.B.J.C.)
† Current address: Department of Immunology, Leiden University Medical Center, Leiden, 2300 RC, The Netherlands
‡ These authors contributed equally to this work.

Citation: Neys, S.F.H.; Heukels, P.; van Hulst, J.A.C.; Rip, J.; Wijsenbeek, M.S.; Hendriks, R.W.; Corneth, O.B.J. Aberrant B Cell Receptor Signaling in Naïve B Cells from Patients with Idiopathic Pulmonary Fibrosis. *cells* **2021**, *10*, x. https://doi.org/10.3390/cells10061321

Academic Editor: Stephen Yarwood

Received: 31 March 2021
Accepted: 20 May 2021
Published: date

Publisher's Note: MDPI stays neutral with regard to jurisdictional claims in published maps and institutional affiliations.

Copyright: © 2021 by the authors. Licensee MDPI, Basel, Switzerland. This article is an open access article distributed under the terms and conditions of the Creative Commons Attribution (CC BY) license (http://creativecommons.org/licenses/by/4.0/).

Table S1. Antibodies used for surface and intracellular staining

Marker	Label	Clone	Company
Human TruStain FcX	-	-	Biolegend
CD19	FITC	HIB19	BD Biosciences
CD19	AF700	HIB19	Invitrogen
CD3	AF700	UCHT1	Invitrogen
BTK	PE	REA367	Miltenyi
CD86	BV650	FUN-1	BD Biosciences
CD38	BV785	HIT2	Biolegend
IgM	Biotin	G20-127	BD Biosciences
IgD	PE-Cy7	IA6-2	BD Biosciences
BAFFR	PerCP-Cy5.5	11C1	Biolegend
CD27	BV421	M-T271	BD Biosciences
Fixable Viability Dye	eFluor506	-	Invitrogen
TACI	APC	1A1	Biolegend
Streptavidin	APC-ef780	-	Invitrogen

Table S2. Antibodies used for phosphoflow cytometry

Marker	Label	Clone	Company
Human TruStain FcX	-	-	Biolegend
CD19	FITC	HIB19	BD Biosciences
CD3	AF700	UCHT1	Invitrogen
CD38	BV785	HIT2	Biolegend
CD27	BV421	M-T271	BD Biosciences
IgD	BV605	IA6-2	BD Biosciences
IgM	Biotin	G20-127	BD Biosciences
Fixable Viability Dye	eFluor506	-	eBioscience
Streptavidin	APC-ef780	-	Invitrogen
pBTK Y223	AF647	N35-86	BD phosflow
pSYK Y348	PE	I120-722	BD phosflow
pPLCγ2 Y759	AF647	K86-689.37	BD phosflow
pPI3K p85 Y458	-	E3UI1H	Cell Signaling Technologies
Donkey-anti-rabbit	PE	-	Jackson ImmunoResearch

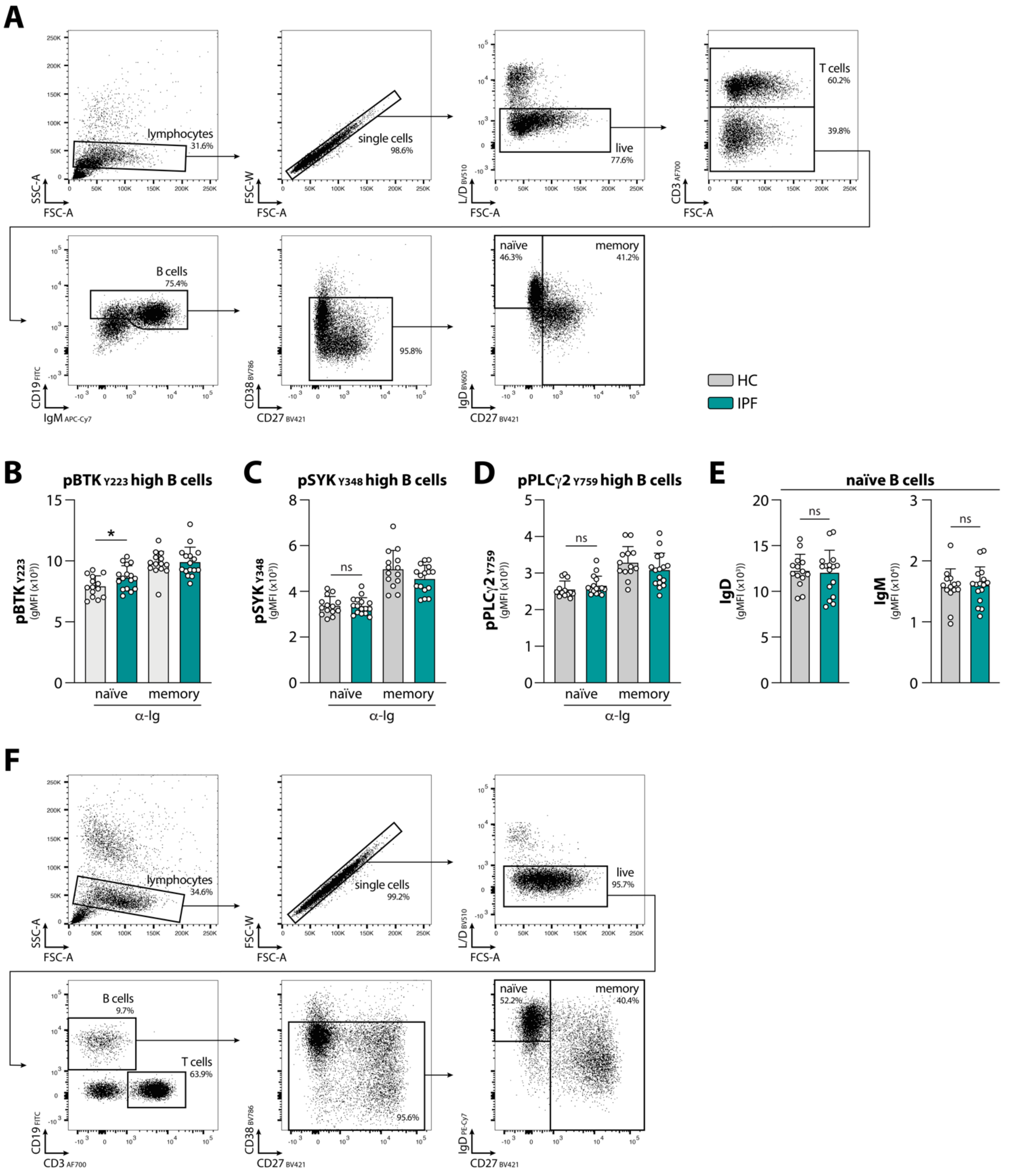

Figure S1. Flow cytometric gating strategies and expression profiles. (**A**) Strategy used for phosphoflow cytometry analysis of the indicated subpopulations. (**B**–**D**) gMFI of pBTK-, pSYK-, and pPLCγ2-high naïve (CD3$^-$CD19$^+$CD38^{lo}CD27$^-$IgD$^+$) and memory (CD3$^-$CD19$^+$CD38^{lo}CD27$^+$) stimulated B cells from HCs and IPF patients (cohort 1). * $p < 0.05$ by an unpaired two-tailed Student's *t*-tests. (**E**) BCR expression on naïve B cells, showing IgD (*left*) and IgM (*right*) surface expression, in HCs and IPF patients (cohort 1). Subjects are indicated by individual data points and bars indicate mean values + SD. (**F**) Strategy used for the analysis of surface and intracellular markers of the indicated subpopulations. SSC-A, side scatter area; FSC-W, forward scatter width; FSC-A, forward scatter area; gMFI, geometric mean fluorescence intensity; L/D, live-dead marker; α-Ig, anti-immunoglobulin; ns, not significant.

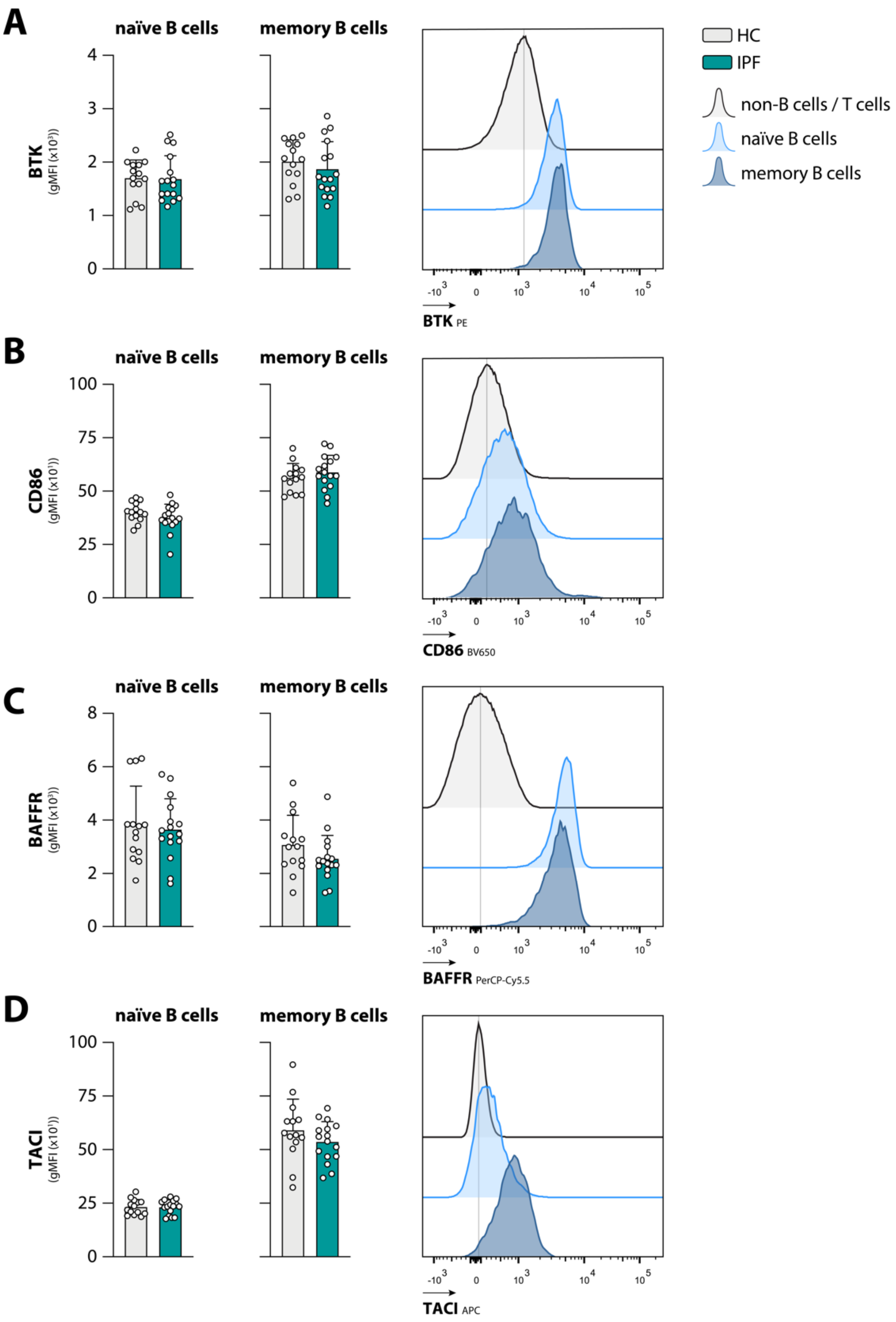

Figure S2. Expression of BTK, CD86, BAFFR and TACI in HC and IPF B cells. Intracellular expression of (**A**) BTK, and surface expression of (**B**) CD86, (**C**), BAFFR, and (**D**) TACI in HC and IPF (cohort 1) naïve and memory B cells (*left*). A representative histogram for non-B cell / T cell, naïve (CD3⁻CD19⁺CD38^lo^CD27⁻IgD⁺), and memory (CD3⁻CD19⁺CD38^lo^CD27⁺) B cell populations from a HC is shown (*right*). Subjects are indicated by individual data points and bars indicate mean values + SD. Differences between IPF patients and HCs were not significant by an unpaired two-tailed Student's *t*-test or Mann-Whitney *U*.

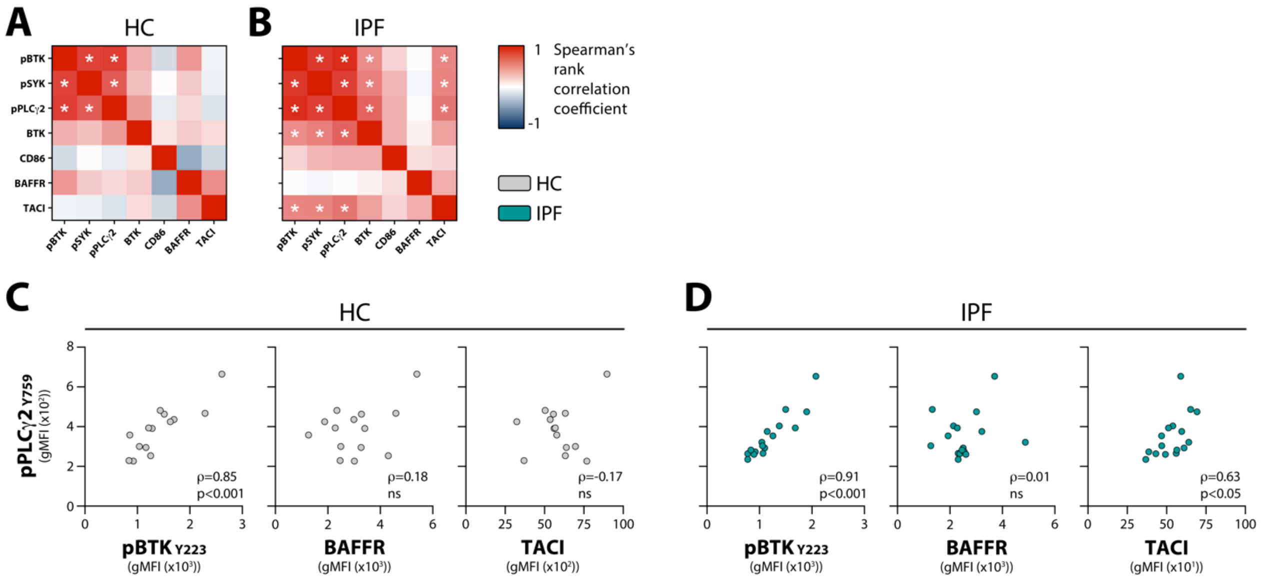

Figure S3. Correlation analysis of ex vivo measured B cell markers and in vitro measured phosphorylation of BCR signalosome molecules in unstimulated memory ($CD3^-CD19^+CD38^{lo}CD27^+$) B cells from HCs and IPF patients (cohort 1). (**A**, **B**) Spearman's rank correlation matrix for (**A**) HCs and (**B**) IPF patients for the indicated markers measured ex vivo and phosphorylated proteins of unstimulated memory B cells. (**C**, **D**) Spearman's rank correlation analysis of (**C**) HCs and (**D**) IPF patients for the correlation of pPLCγ2 with pBTK, BAFFR, and TACI expression in unstimulated memory B cells. Subjects are indicated by individual data points. ns, not significant.

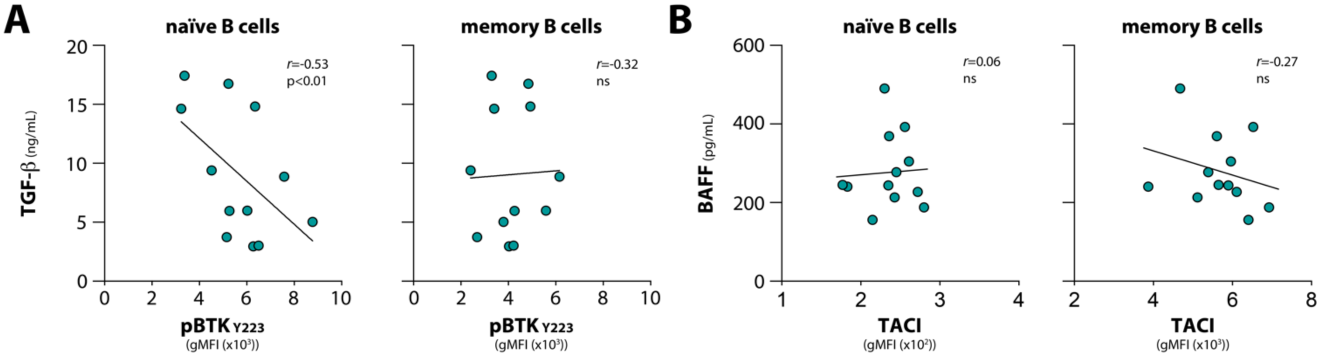

Figure S4. Correlations of circulating cytokines in IPF patients (cohort 1). (**A**) Correlation of circulating TGF-β levels and phosphorylation of BTK following α-Ig stimulation in naïve ($CD3^-CD19^+CD38^{lo}CD27^-IgD^+$) and memory ($CD3^-CD19^+CD38^{lo}CD27^+$) B cells from IPF patients. (**B**) Correlation between circulating BAFF levels and TACI expression on naïve or memory B cells from IPF patients. Subjects are indicated by individual data points. ns, not significant.

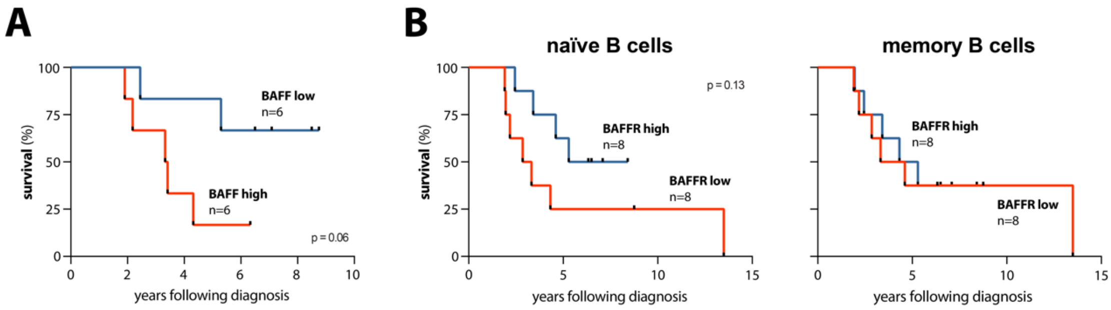

Figure S5. Survival analysis for IPF patients based on circulating BAFF levels and BAFFR surface expression. IPF patients (cohort 1) were ranked from highest to lowest BAFF level or BAFFR surface expression and subsequently split into two equal groups. Survival Kaplan-Meier curve for (**A**) circulating BAFF levels and (**B**) naïve ($CD3^-CD19^+CD38^{lo}CD27^-IgD^+$) and memory ($CD3^-CD19^+CD38^{lo}$ $CD27^+$) B cell surface BAFFR expression. Data were analyzed using a log rank test for statistical significance.
